# Characteristics of the sleep structure in the cleaner wrasse *Labroides dimidiatus*

**DOI:** 10.1186/s40851-025-00258-z

**Published:** 2026-01-08

**Authors:** Masayuki Yoshida, Atsuki Izumi, Shumpei Sogawa, Satoshi Awata, Masanori Kohda

**Affiliations:** 1 Graduate School of Integrated Sciences for Life, Hiroshima University, Higashihiroshima, 739-8528 Japan; 2 Laboratory of Animal Sociology, Department of Biology, Graduate School of Science, Osaka Metropolitan University, Osaka, 558-8585 Japan

**Keywords:** Behavioral measurements, Cleaner wrasse, REM-Non-REM sleep, Sleep architecture, Sleep in fish

## Abstract

Sleep is a widespread phenomenon among animals, yet its evolutionary traits and core functions remain largely enigmatic. To elucidate the fundamental characteristics of behavioral sleep in fish, we conducted quantitative assessments of behavioral and physiological properties, including body movement, eye movement, yawning, and ventilation, during sleep in the cleaner wrasse *Labroides dimidiatus* under laboratory conditions. The sleep states of the cleaner wrasse were characterized by a decreased ventilation rate, occasional distinctive waving movements, rapid eye movement (REM) episodes, and an increasing trend in both ventilation rate and REM episodes toward the end of the night period. Waving movements, temporal decreases in ventilation rate, and REM episodes showed distinct relationships. The patterns of behavioral and physiological features observed in the cleaner wrasse closely resemble those documented in mammals, leading us to propose that the structure of behavioral sleep is conserved across vertebrate species. Our findings further support the notion that the alternation of two  sleep states, Non-REM/slow-wave sleep and REM/paradoxical sleep, constitutes a shared sleep structure of sleep across a wide range of vertebrate species.

**Supplementary Information:**

The online version contains supplementary material available at 10.1186/s40851-025-00258-z.

## Background

Sleep is a phenomenon observed across a wide range of animal taxa, from cnidarians to mammals [1–5]. The behavioral state of sleep is characterized by several criteria: (1) circadian-regulated behavioral quiescence or minimal motor activity; (2) an elevated sensory threshold for arousal; (3) species-specific postures and place preferences; and (4) homeostatic regulation, wherein sleep deprivation leads to a compensatory increase in sleep duration [1, 2, 5–9]. Although many chondrichthyan and actinopterygian fish species exhibit sleep-like resting behaviors that meet the first two or three criteria, only a limited number of fish species satisfy all four criteria [9]. Determining sleep homeostasis is compounded by the challenges of designing experiments that require selecting ethologically relevant stimuli to disrupt the sleep state without imposing undue stress on the fish. Fish represent the largest group of vertebrates and occupy a significant position in the animal phylogeny. Despite this significance, they have been largely overlooked in sleep studies. Sleep behavior in fish shows great variability influenced by their diverse habitats and ecological contexts [10].

In the field of sleep research, polysomnography (PSG) has a well-established history in the monitoring of sleep characteristics, including muscular, cardiac, ocular, and various other physiological and motor activities, in conjunction with electroencephalogram (EEG) recordings during sleep [7, 11–13]. This technique has helped elucidate that sleep is divided into two primary states: Non-REM/Slow wave sleep (Non-REM/SWS), characterized by synchronized slow cortical activity and the absence of rapid eye movement (REM), and REM/paradoxical sleep (REM/PS), distinguished by atonia and the presence of REMs. The alternation between Non-REM/SWS and REM/PS has been observed across a range of mammalian, avian, and reptilian species [1, 3, 7, 14–17]. Recent studies have further identified neural signatures of sleep in larval zebrafish, where brain-wide neural activity and other physiological activities were monitored optically [18]. This study identified two types of sleep dynamics: slow bursting sleep (SBS) and propagating wave sleep (PWS), which are proposed to correspond to Non-REM/SWS and REM/PS, respectively. However, despite many features of PWS aligned with the criteria for REM/PS, PWS lacks rapid eye movements, which are hallmarks of REM sleep. The absence of rapid eye movements during sleep has also been documented in adult zebrafish [19] and the cichlid *Tilapia mossambica* (now known as *Oreochromis mossambicus*) [20]. Conversely, observations of tropical marine fishes have revealed periodic eye movements during nocturnal rest, suggesting the potential existence of a REM sleep state in certain fish species [21, 22]. Comprehensive and quantitative investigations of the architecture of behavioral sleep in representative fish species are necessary to elucidate the biological mechanisms and evolutionary aspects of sleep in vertebrates.

Although the fourth criterion of behavioral sleep, sleep homeostasis, has not been systematically investigated, numerous observations in both wild and laboratory settings have demonstrated that diurnal Labridae wrasses engage in sleep during the night in specific “beds”, such as under rocky shelters and sandy bottoms [21–23]. Among these observations, qualitative assessments of behavioral characteristics during sleep have been documented for the cleaner wrasse *Labroides dimidiatus* [21]. It has been reported that the cleaner wrasse remains in a shelter throughout the dark period, whereas resting on the bottom of the tank or in the shelter during the light period has rarely been observed under laboratory conditions [21]. This distinct diurnal activity pattern, particularly the consistent presence in the shelter during the night period, renders this species suitable for a detailed examination of subtle behavioral events during behavioral sleep in laboratory conditions. Furthermore, the advanced cognitive abilities and social interactions of the cleaner wrasse warrant a comprehensive investigation into the relationships between the cognitive processes and sleep architecture of this species [24, 25]. Although sleep homeostasis of the cleaner wrasse is yet to be determined, the negative effects of sleep disruption on cognitive functions, such as learning, have been demonstrated in this species, suggesting that the role of sleep in mental processes is shared among vertebrates [26].

The present study aimed to describe the detailed features of behavioral sleep in the cleaner wrasse and compare them with other vertebrates to gain further insight into sleep behavior and its evolution. In this study, using video analysis of laboratory-kept cleaner wrasse, we quantified body movements, eye movements, and mouth opening and closing during the nocturnal resting period when the fish remained in a shelter. Additionally, we measured the ventilation frequency based on opercular movement as a physiological parameter alongside behavioral measurements.

## Methods

### Subject fish

Wild-captured adult cleaner wrasse (*Labroides dimidiatus*) were obtained from commercial sources. This is a small protogynous hermaphrodite fish, up to 15 cm in total length (TL), changing sex from female to male. The size of the subject fish was 7–8 cm and would be functionally females [25]. They were housed individually in laboratory aquaria (45 × 30 × 28 cm) for a minimum of one week prior to experimentation. The fish were maintained under a constant 12:12-hour light:dark cycle at a water temperature of approximately 25 °C. They were fed a small portion of shrimp abdominal meat once daily.

All animal experiments were conducted in accordance with the Guidelines for Animal Experimentation of Hiroshima University (approval number F23–3) and Osaka Metropolitan University (approval number S0088).

### Video recording

Aquaria used for acclimating the fish were employed for the study. The transitions between light and dark were gradually adjusted over a 30-minute period before complete cessation or initiation of illumination. Consequently, the light, dimming, and dark periods lasted 11, 1, and 12 h, respectively. A semicylindrical plastic shelter (98–110 mm in length, depending on the size of the subject fish) was affixed to the side of the aquarium with its interior visible from outside the aquarium. A visible-light cut and near-infrared-light transmissive plastic plate was installed on the exterior of the aquarium at the shelter’s location to minimize visual interference with the fish inside the shelter. Two near-infrared (NIR) light (wavelength, 940 nm) emitters (S20D-IR, Energypower, Tokyo, Japan) and an infrared-light video camera (DMK23UM021, The Imaging Source, Bremen, Germany) connected to a computer (ExpertBook, ASUS, Taipei, Taiwan) via a USB were positioned in front of the observation aquarium.

Following the setup of the experimental apparatus, the fish were acclimated for an additional two-three days to ensure that they rested in the shelter throughout the dark period. Although the fish occasionally entered the shelter during the light period, these stays were brief and lasted less than a few minutes. Video recording commenced one hour before the onset of the dark period and concluded one hour after the end of the dark period. Recordings were conducted over four to five consecutive days for five fish. Video quality was critical for subsequent quantitative analysis. Two recordings from fish #1, one from fish #2, three from fish #3, two from fish #4, and one from fish #5 were considered satisfactory and were subjected to further analysis. Because the latter half of one recording from fish 1 was inadequate for behavior quantification, it was excluded from certain analyses.

Behavioral parameters, such as the movements of the eyes, operculum, mouth, and trunk, were tracked by obtaining the coordinates of various body parts of the fish from video recordings at a rate of 40 fps using DeepLabCut [27]. For eye-movement tracking, the coordinates of the dorsal, ventral, rostral, and caudal margins of the eye, as well as the center of the pupil, were recorded. Opercular movement was monitored by registering the coordinates of five equally spaced points on the lid. Whole-body movement was assessed by recording the coordinates of the four points on the body stripe. In addition, the coordinates of the tips of the upper and lower lips were recorded. The rostral end of the stripe served as a reference point to subtract the positional change of the fish in the video frame from the coordinates of other points. LabChart software (ADInstruments, Bella Vista, Australia) and proprietary software developed in LabVIEW 2023 (NI, Austin, TX, USA) were employed to calculate and quantify bodily movements.

Eye movements were quantified by calculating the horizontal and vertical rotations of the eye. The measures were integrated and thresholded to detect the occurrence and duration of the REM episodes. Ventilation activity was deduced from fluctuations in the arch shape formed by five points on the opercular lid. Peaks of these fluctuations were detected, and the instantaneous frequency of the ventilation rate was calculated. In instances where ventilation frequencies were assessed just after entering and just before exiting the shelter, the number of opercular expansions and compressions were manually counted for 15–25 s, with the duration varying depending on video quality, in each period. Body bending or waving (see the results for details) was detected by monitoring the changes in distances between points on the stripe. When peri-event analysis was required, the waving movement was integrated to obtain the amplitude of movement. The distance between the tips of the upper and lower lips was considered to reflect mouth opening-closing movements. Extraordinarily wide openings in the mouth were identified as “yawning” (see Fig. 2). This activity was clearly distinguished from ventilation-related mouth opening-closing, which was small and often undetectable. All measures, except ventilation frequency, were standardized for each fish for further analysis.

### Statistics

All statistical analyses were conducted using the statistical platform R version 4.4.1 [28]. To assess whether the behavioral parameters changed during the 12 h dark periods, we performed linear mixed models (LMMs) or negative binomial generalized linear mixed models (GLMMs) (R packages lme4 and lmerTest). In all these models, individual ID was included as a random effect to account for the repeated-measures design (*n* = 8 or 9 observations from five fish). Changes in ventilation frequency (Hz) and the proportion of REM duration in 1-hour time blocks during the 12 h dark periods were assessed using LMMs. Temporal changes in yawning frequency (/h) and waving frequency (/h) were analyzed using negative binomial GLMMs. Significant effects were determined using the ANOVA function (R package car, type II Wald χ^2^ test). Statistical significance was set at *p* < 0.05.

## Results

### Typical sleeping behavior

**Fig. 1:**
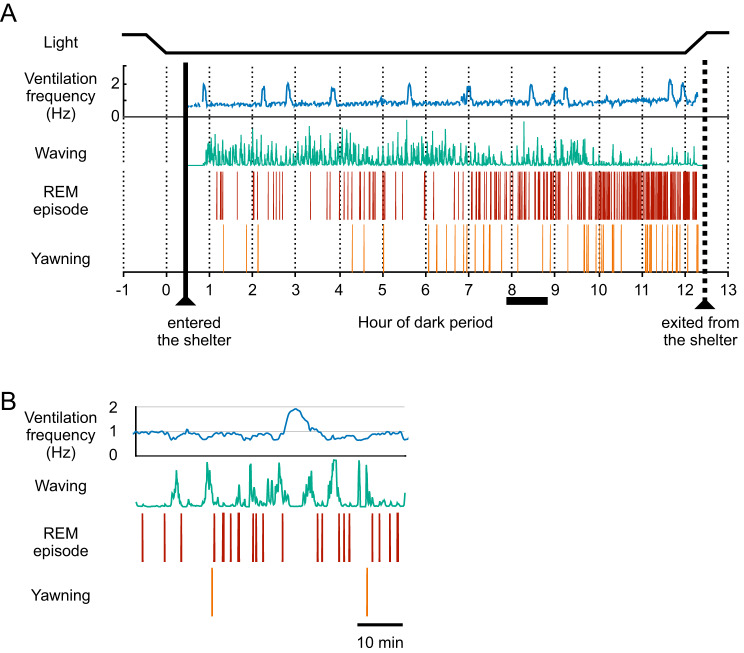
An ethogram representing the sleep behavior of an individual cleaner wrasse is presented. (**A**) the overnight ethogram illustrates the ambient light conditions, ventilation frequency, waving, REM episodes, and yawning. The ventilation frequency was smoothed over a 1.5-minute interval. The waving movement is depicted as an integrated and standardized waveform to indicate its timing and intensity. REM episodes and yawning are marked with tick marks to denote their occurrence. The thick vertical solid and dashed lines represent the times when the fish entered and exited the shelter, respectively. (**B**) an expanded time scale of the ethogram for the period indicated by the thick horizontal bar in A

During the light period, the cleaner wrasse exhibited continuous swimming behavior within the laboratory aquarium. In contrast, the subject fish entered the shelter shortly after the cessation or dimming of ambient illumination, facilitating uninterrupted observation of their bodily movements. The behavioral sleep of the cleaner wrasse was characterized by diminished responsiveness to external stimuli, such as vibration due to door opening—closing and transient illumination by small flashlights, and minimal movement of the pectoral fins, which are typically active during daylight hours. All the fish observed in the current experiment remained within the shelter throughout the dark period, without exception. However, in the preliminary experiments, some fish occasionally exited the shelter and returned after a brief interval. Figure 1 presents a representative overnight ethogram illustrating four parameters: ventilation frequency, waving movement, REM episodes, and yawning. The general characteristics of these measures include both regular and irregular fluctuations in ventilation frequency, periodic occurrence of waving movements, and an increase in REM and yawning episodes towards the conclusion of the dark period.

**Fig. 2:**
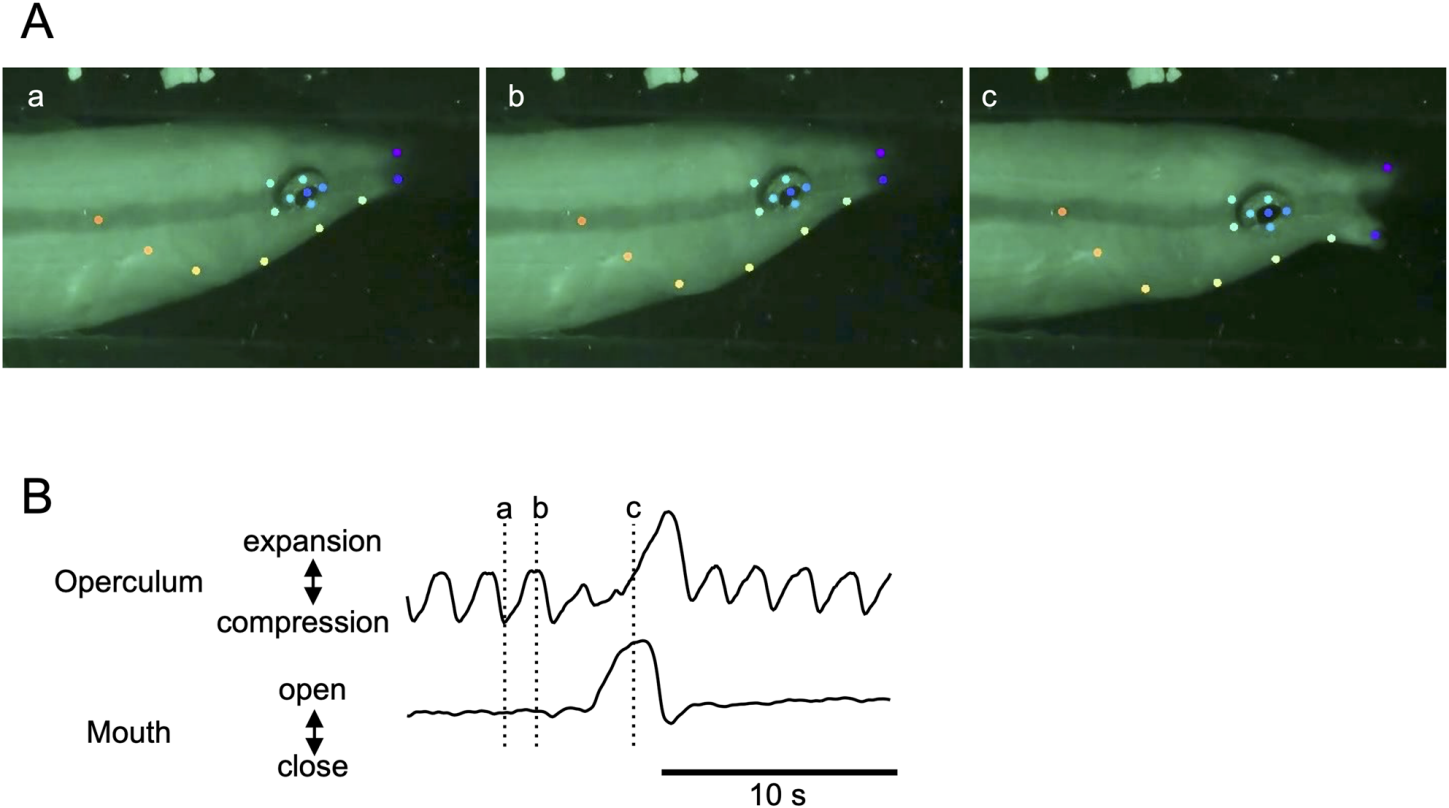
Ventilation and yawning behavior in a cleaner wrasse. (**A**) extracted video frames illustrate the operculum in a compressed state with the mouth partially closed (Aa), operculum expanded with the mouth partially closed (Ab), and operculum partially expanded with the mouth fully open, indicating a yawn (Ac). Colored dots represent specific body locations identified by the DeepLabCut software (refer to the text for further details). (**B**) waveforms depicting operculum expansion and compression to visualize ventilatory movements (upper trace) and mouth opening and closing to detect yawning (lower trace). The vertical dotted lines, labeled a, b, and c, correspond to the timings of video frames a, b, and c in a, respectively

### Ventilation and yawning

**Fig. 3:**
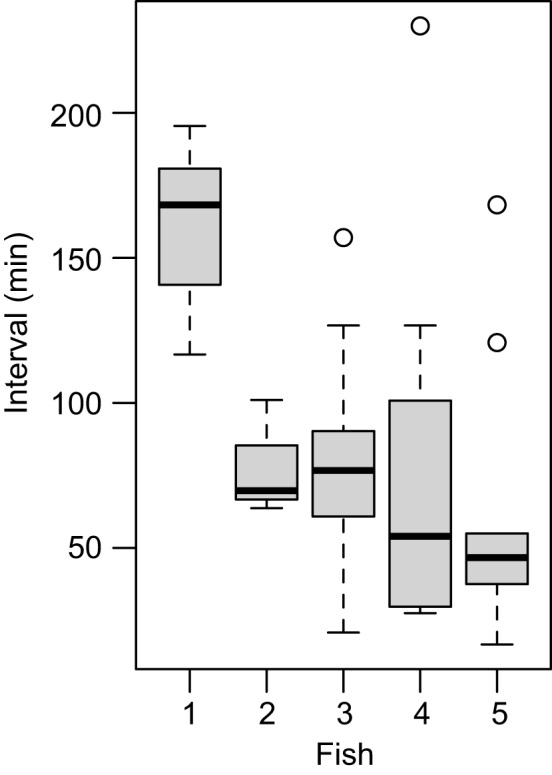
Box plots illustrating the intervals of high-frequency ventilation episodes during the nocturnal period in five individual cleaner wrasse

**Fig. 4:**
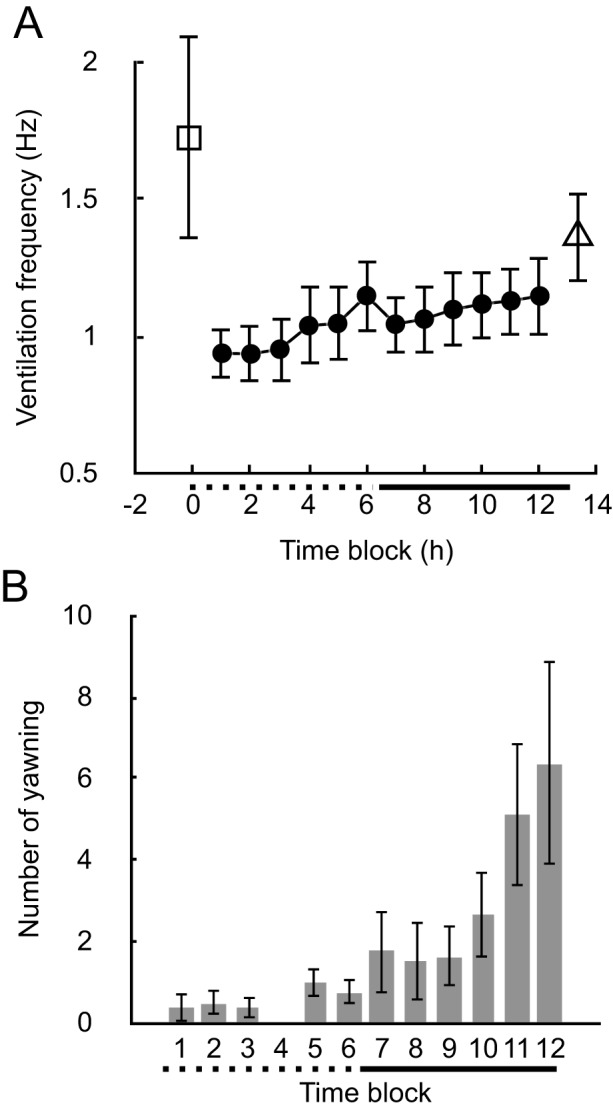
Ventilation rate (**A**) and yawning frequency (**B**) of cleaner wrasse during the night period. (**A**) Filled circles represent the mean ventilation frequencies in 1-hour time blocks. The open rectangle and triangle denote the mean ventilation frequency immediately after entering and just before exiting the shelter, respectively. (**B**) The bar graph illustrates the mean number of yawns in 1-hour time blocks. Dotted and solid horizontal lines indicate the first and the latter half of the dark period, respectively. Bars indicate standard error of the mean (SEM). *n* = 8 nights from 5 fish

Ventilation activity was not consistently associated with apparent rhythmic mouth opening and closing; therefore, the ventilation frequency was quantified by observing the expansion and compression of the operculum (Fig. 2). Occasionally, wide mouth openings, termed yawning, were observed (Fig. 2A). Mouth opening was followed by a marked expansion of the operculum, suggesting that increased water flow through the gills accompanies these yawning activities (Fig. 2B). At times, ventilation frequency exhibited phasic increases to approximately 2 Hz, lasting several minutes (Fig. 1). The periodicity of this event was not evident, although the interval range was approximately 60 min and varied among individual fish (Fig. 3). No apparent correlation was found between this temporal increase in ventilation and other measures. The average ventilation frequency immediately after entering the shelter was 1.721 ± 0.371 Hz (mean ± SEM) (Fig. 4A). This frequency rapidly decreased, with an average frequency of 0.938 ± 0.096 Hz (mean ± SEM) during the first hour, and remained low for several hours. An increasing trend in basal ventilation frequency toward the end of the sleep period was evident (Figs. 1, 4).

A comparison of basal ventilation frequency, excluding episodes of high ventilation frequency, from the beginning to the end of the sleep period revealed a significant increase in basal ventilation frequency toward the end of sleep (LMM, Wald χ^2^ = 19.90, df = 11, *p* = 0.047) (Fig. 4A). The ventilation frequency immediately after entering the shelter was 1.721 ± 0.371 Hz (mean±SEM) (Fig. 4A). The frequency increased rapidly just before exiting the shelter to 1.363 ± 0.164 Hz (mean±SEM), which was not statistically different from the frequency immediately after entering the shelter (paired t-test, *t* = 0.694, df = 4, *p* = 0.526), although the level remained low (Fig. 4A). Yawnings were scarce during the first half of the dark period, showing an increasing trend toward the end of the dark period (negative binomial GLMM, Wald χ^2^ = 88.51, df = 11, *p* < 0.0001) (Fig. 4B).

### Waving

**Fig. 5:**
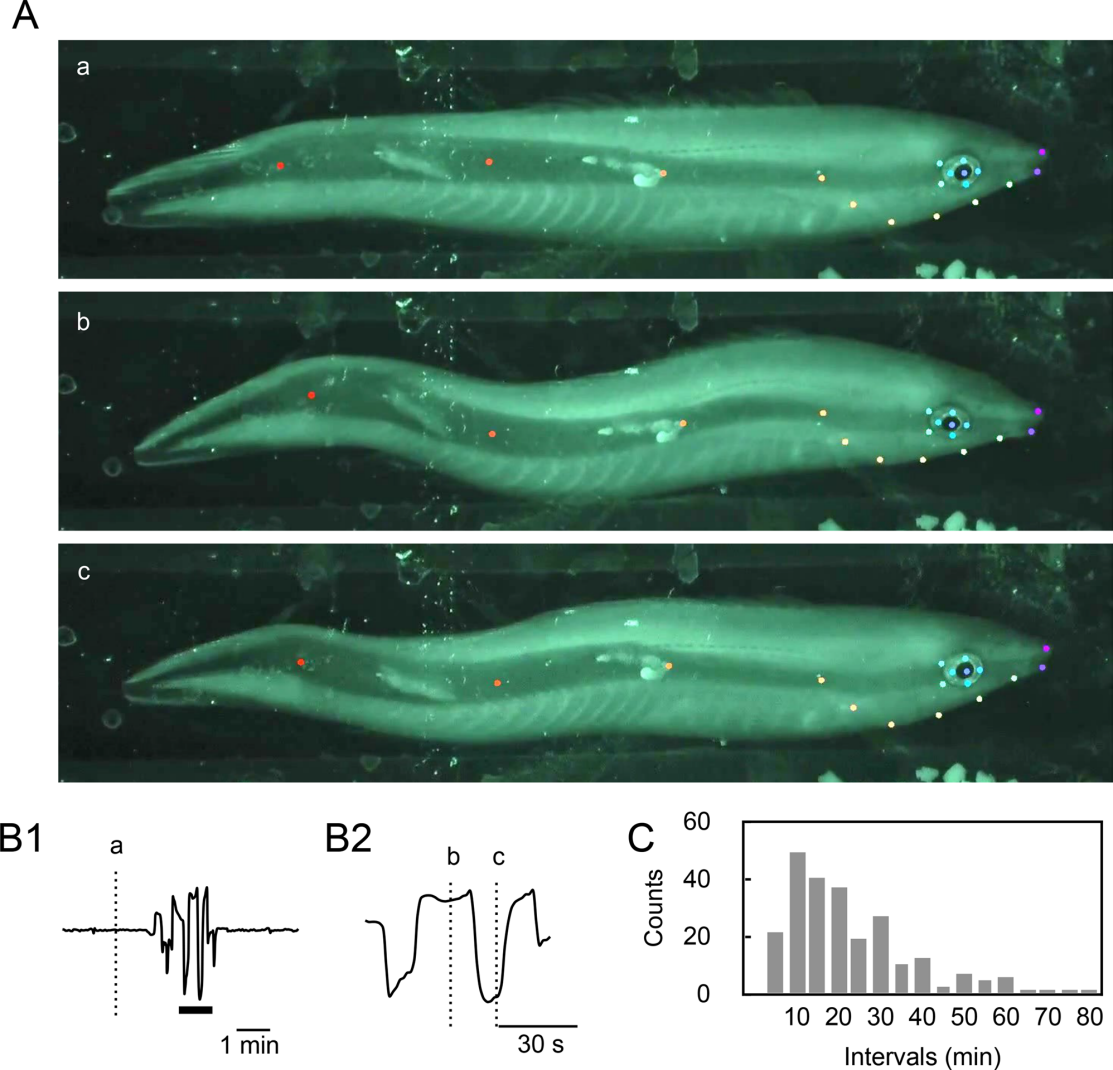
Waving behavior of cleaner wrasse. (**A**) Extracted video frames depicting alternating trunk bending. (Aa) Prior to waving. (Ab) Wave-formed bending of trunk. (Ac) Wave-formed bending of the trunk to the opposite side. Colored dots indicate specific body locations identified by the DeepLabCut software. (**B1**) Time course of waving behavior illustrating alternating wave-formed bendings. (**B2**) Expanded timescale of the period denoted by the thick horizontal bar in B1. Note that the fish remained bent for a period before bending to the opposite side. Vertical dotted lines, labeled a, b, and c in B1 and B2, correspond to the timing of video frames a, b, and c in A, respectively. (**C**) Histogram illustrating the frequency of waving behavior occurrences at various intervals. *n* = 8 nights from five fish

A distinctive bodily movement observed during sleep was slow undulation of the trunk (Fig. 5A). Unlike the undulating locomotory movement observed during the daytime, this movement did not appear to generate propulsive force for locomotion. The trunk bent into a wave shape and maintained this form momentarily before bending to the opposite side, with this movement alternating for a few minutes (Fig. 5B). During the behavioral sleep of each subject fish, 31.5 ± 4.309 (mean±SEM, *n* = 8 from 5 fish) episodes of waving movement were recorded. The number of waving episodes in the first half of the dark period was 18.25 ± 2.624 (mean±SEM, *n* = 8 from 5 fish), which was significantly higher than in the latter half, 12.875 ± 2.083 (mean±SEM, *n* = 8 from 5 fish, negative binomial GLMM, Wald χ^2^ = 23.13, df = 11, *p* = 0.017). The major intervals of the waving episodes ranged from 10 to 30 min (Fig. 5C). This behavior was exclusively observed during the dark period and absent during the active light period.

### Rapid eye movements

**Fig. 6:**
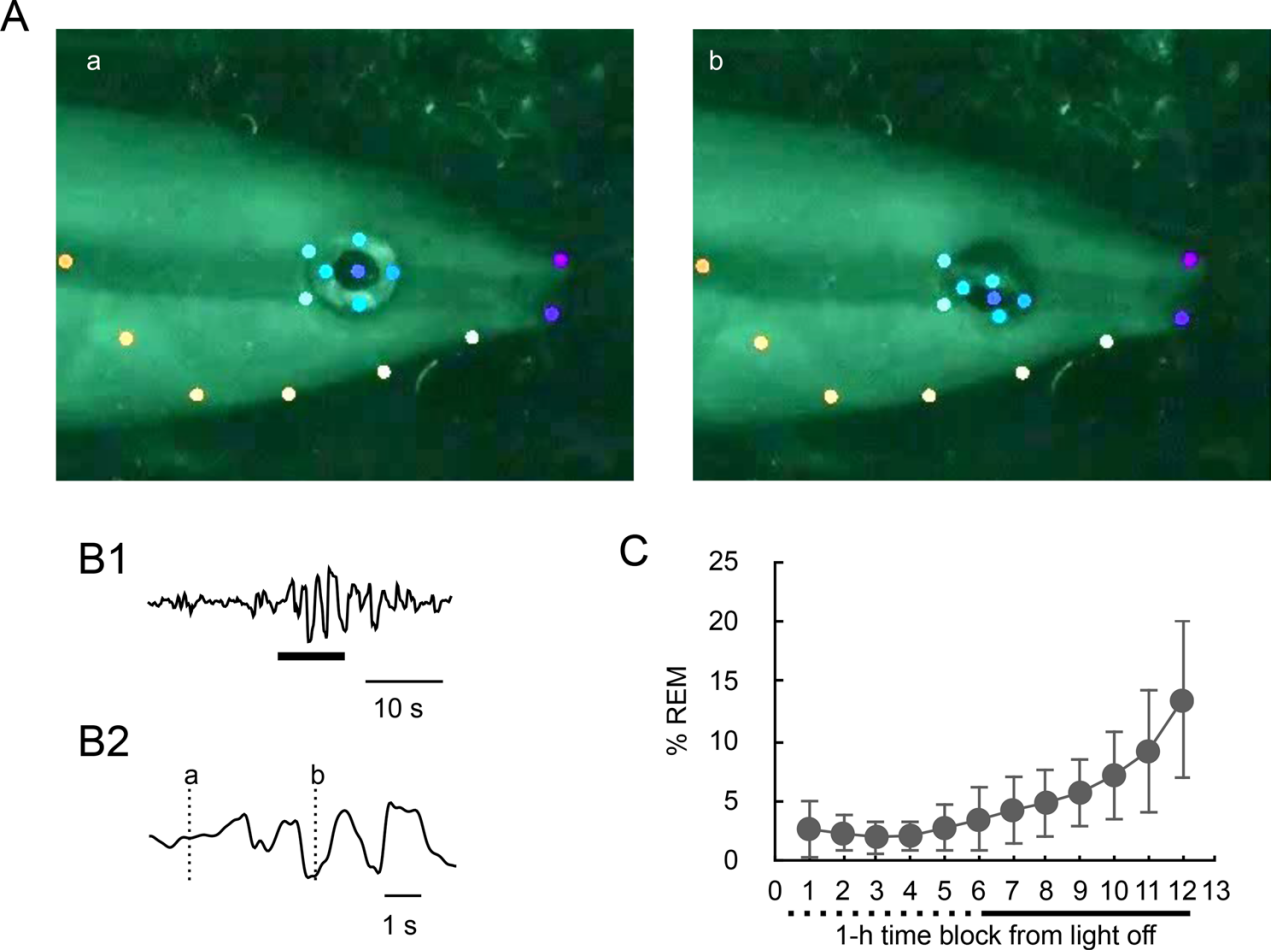
Rapid eye movement (REM) in cleaner wrasse. (**A**) Extracted video frames depicting rapid eye movement (REM in an individual specimen. Colored dots indicate specific body locations identified by DeepLabCut software. (Aa) Moment immediately preceding REM. (Ab) Downward eye rotation during the REM episode. (**B1**) Waveform indicating the vertical movement of the eye during the REM episode shown in A. (**B2**) Expanded time scale for the period denoted by a thick horizontal bar in B1. Vertical dotted lines, labeled a and b in B2, correspond to the timings of the video frames a and b in A. (**C**) Proportion of REM episodes in 1-hour time blocks over the dark period. Dotted and solid horizontal lines indicate the first and the latter half of the dark period, respectively. Means±SEMs are presented. *n* = 8 nights from 5 fish

REMs were observed as occasional twitch-like movements during sleep (Figs. 1, 6A,B). The frequency of REMs was relatively low during the first half of the dark period and increased towards the end of the period (Figs. 1, 6C) across all fish observed, although the abundance of REM episodes varied among the individuals. Figure 6C illustrates the proportion of REM duration in 1-hour time blocks during the dark period. The proportion of REM was less than 5% in the first half of the dark period and markedly increased during the latter half, reaching nearly 15% at the end of the dark period or just before waking (LMM, Wald χ^2^ = 193.72, df = 11, *p* < 0.0001).

### Relationships among measures

**Fig. 7:**
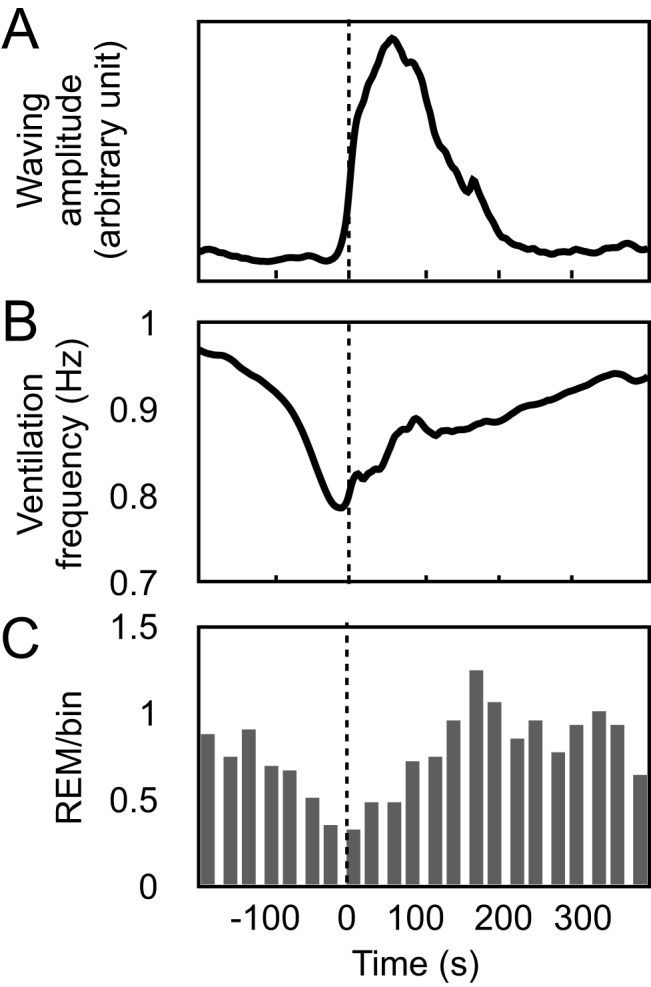
Ventilation frequency and REM occurrence in relation to waving activity. The data represent averages of five individuals. For each fish, waving activities (**A**), as exemplified in B1 of Fig. 5, were standardized and integrated. The progression of ventilation frequency (**B**) and the count of REM occurrences in 20-second intervals (**C**) were then analyzed in relation to the integrated waving movements

Our findings revealed distinct associations among waving behavior, ventilation frequency, and the occurrence of REM episodes (Figs. 1B, 7. Notably, both ventilation frequency and the probability of REM occurrence began to decline 1–2 minutes prior to the initiation of waving events, subsequently returning to baseline levels as waving movements diminished (Fig. 7).

## Discussion

Although numerous species of both chondrichthyan and actinopterygian fish have been documented to meet certain criteria for behavioral sleep [8], detailed information on the behavioral signatures that constitute sleep in fish remains limited [20–22, 29, 30]. Sleep states are also characterized by specific EEG patterns and are divided into Non-REM/SWS and REM/PS. The alternation between Non-REM/SWS and REM/PS is evident in mammals, birds, and reptiles [3]. Recent polysomnographic studies on larval zebrafish, which involve optical brain-wide activity monitoring, have indicated potential counterparts of NonREM/SWS and REM/PS in the neuronal signatures of sleep in zebrafish larvae [18]. Another study on zebrafish also suggested that the neurochemical and neuro-connective bases for regulating sleep are conserved from fish to mammals [31–33]. These findings imply that both behavioral and neuronal sleep states are broadly conserved across vertebrates [3, 18].

Although zebrafish serves as a promising model organism for investigating the neurophysiological, molecular, and genetic aspects of sleep, it is potentially limiting to rely on a restricted number of fish species, especially given the vast diversity of fish adapted to various environments, for studies on behavioral adaptation.

For example, zebrafish exhibit relatively short sleep episodes that predominantly occur at night, interspersed with active wakeful periods [30]. Some sleep bouts also occur during the day [30]. Therefore, non-model fish are crucial for understanding the variation, diversity, and the adaptive and ecological significance of sleep.

Wrasses of the Labridae family, including the cleaner wrasse, are empirically known to exhibit distinct circadian activity-rest cycles and “sleep soundly” throughout the night [21] Additionally, they demonstrate strong place preferences, such as seeking shelter among rocks or reefs and burrowing under sandy bottoms [21, 34, 35]. Consequently, the cleaner wrasse is well suited for close observation of behavioral changes during the night. They are active during the daytime, and resting on the bottom or in shelters during the light period is rarely observed, at least under laboratory conditions [21]. The overnight stable presence of this species in a shelter also facilitates continuous observation and allows for monitoring of ventilation frequency over extended periods.

On the other hand, while the behavioral manifestations of sleep in the cleaner wrasse are empirically evident and partially documented in prior research, confirmation of sleep homeostasis remains elusive. Previous investigations have explored the effects of sleep deprivation induced by nocturnal illumination in this species [26]. Although cognitive function was partially but significantly impaired in the following day because of sleep deprivation, no apparent alterations in sleep behavior were observed after the deprivation [26]. This discrepancy may be attributed to the mildness of the light stimulus, which was insufficient to affect subsequent sleep behavior, despite its significant impact on cognitive tasks. Therefore, the parameters of sleep deprivation studies require thorough examination. The findings of this study are expected to contribute to future study for a deeper understanding of sleep homeostasis in the cleaner wrasse.

In this study, we successfully employed video analysis techniques to continuously monitor multiple behavioral and physiological parameters of cleaner wrasse, which is particularly amenable to close observation during nighttime. Quantitative overnight monitoring of bodily movements, including waving, REM, and yawning, was conducted alongside ventilation activity as a physiological measure.

Waving is characterized as a slow, alternating lateral bending of the trunk, distinct from the undulating movement used for locomotion. This movement pattern was specific to the dark period and was not observed during the active light period. The intervals of occurrence of waving were predominantly 10–30 min, with a significant tendency to be more frequent in the first half of the dark period. Prolonged maintenance of a single posture can lead to muscle stiffness in humans; nonetheless, engaging in regular muscle contractions can alleviate these effects [36]. However, in the case of the cleaner wrasse, this phenomenon is unlikely, as the maintenance of muscular tone for postural support does not appear necessary in an aquatic environment. While the impairment of local blood flow due to pressure on specific tissues is also improbable, it remains possible that periodic contractions of trunk muscles may enhance or support systemic blood flow. Furthermore, it is premature to dismiss the possibility that the cleaner wrasse may experience some form of discomfort due to the static relaxation of muscles during extended postures [37].

Although the purpose of waving remains unclear, correlations were observed among the occurrence of waving, ventilation frequency, and REM episodes. The ventilation frequency decreased tens of seconds prior to the onset of waving, and this decrement coincided with a decrease in the probability of REM episode occurrence. In humans, an inverse movement-REM correlation has been well-documented [15, 38]. The present findings support the idea that the REM episode in cleaner wrasse is also associated with atonia, similar to that in mammals. In humans, respiration is faster and more irregular during REM sleep than during SWS [15, 38, 39]. The present findings regarding the correlations among waving, ventilation frequency, and REM episodes align with the characteristics of the human sleep architecture.

An increasing trend toward awakening of the proportion of REM duration was also evident. However, it remains to be determined whether this suggests that the behavioral phenotypes of sleep are shared across a broad range of vertebrates, as some fish species examined thus far do not exhibit apparent REM during sleep [18–20]. It is premature to conclude that REM in cleaner wrasse represents the REM/PS state observed in terrestrial vertebrates. Nevertheless, considering the findings of this study along with recent optical PSG research in zebrafish, the alteration between Non-REM/SWS and REM/PS sleep is highly probable across vertebrates [3, 18]. Further investigation of the neural and endocrinological basis of these prominent behavioral features in fish sleep could significantly contribute to understanding sleep dynamics and its evolution in animals. Recent findings have suggested that there are two alternating states of sleep, which might correspond to REM-Non-REM sleep, even in some invertebrate species, including the cephalopod *Octopus* [40] and an arthropod *Drosophila* [41]. We might need to reconsider the criteria for characterizing sleep architecture to extend our view to better understand the function and evolution of sleep in animals.

During sleep, the cleaner wrasse exhibited two notable changes in ventilation activity. First, there is a periodic increase in ventilation frequency that occurs approximately every hour, lasting for a few minutes. During this period, the ventilation frequency reached levels comparable to those observed immediately after the fish entered the shelter, suggesting partial arousal. It is also possible that this temporary increase in ventilation frequency serves as a compensatory mechanism to enhance gas exchange in the gills, particularly in shelters where water may become stagnant [42]. While increased respiration during REM/PS has been documented in humans [15], cleaner wrasse does not exhibit this pattern. Second, there is a significant trend of increasing baseline ventilation frequency towards the end of the dark period. Initially, ventilation frequency decreased rapidly when the fish entered the shelter at the onset of the dark period. However, after a few hours, it gradually increased and returned to pre-rest levels by the end of the dark period. This observation suggests that the deepest sleep in the cleaner wrasse occurred during the first three hours of the dark period. It has been established that respiration rate is influenced by circadian rhythms and sleep-wake states in rats and mice, with respiration increasing towards the beginning of the active phase [43–45]. The increase in ventilation frequency in the cleaner wrasse towards the end of the dark period may also be regulated by circadian rhythms in preparation for anticipated metabolic demands. Conversely, it has been reported that this species exhibits a marked decrease in responsiveness to external stimuli only after 2–4 hours of the dark period [21]. In the present study, no artificial stimuli were applied to the fish to observe and describe their normal sleep behavior. The relationship between physiological responses such as ventilation and behavioral responses to external stress during sleep in fish remains to be elucidated.

Yawning is a fixed action pattern observed across various vertebrate groups [46, 47]. In fish, as in other vertebrates, yawning plays a preparatory role in increased activity and may enhance physiological arousal [46–48] Moreover, in both mammals including humans, and fish, yawning is associated with transitions in behavioral states, such as from resting to active, or precedes activity upon awakening [46, 48–52]. The significant increase in yawning near the end of the dark period in the cleaner wrasse supports the hypothesis that yawning serves a preparatory function in transitioning to an active state before dawn. Thus, yawning appears to have two functions in fish: one is physiological and behavioral at the individual level, and the other is social, as reported for zebrafish, where yawning plays a role in synchronizing motor actions within social groups [47, 48]. Yawning was also observed at a low frequency at midnight in cleaner wrasse. It is possible that these yawns are associated with temporary and/or partial awakening at night. However, no clear correlation was found between yawning and the other events. In addition, considerable individual differences also limit further discussion on the function of midnight yawning.

It is important to acknowledge certain limitations of the current observations of behavioral sleep in a cleaner wrasse. One significant limitation is the potential influence of the laboratory environment on the subjects’ behavior. Notably, the absence of social interactions, both intra- and inter-species, under laboratory conditions during the daytime may impact the sleep behavior of this highly social fish species. Future research should focus on examining the effects of social interactions on sleep behavior in this species to further elucidate the biological characteristics of sleep behavior.

## Conclusion

Through a comprehensive quantitative analysis of sleep behavior in the cleaner wrasse, we propose that the structure of behavioral sleep, as also evidenced by neural and hormonal signatures [18, 30–33, 53], is conserved across a broad spectrum of vertebrate species. The patterns of whole-body movement, yawning, and REM observed in the cleaner wrasse closely resemble those documented in mammals, including humans. Furthermore, the correlation between ventilation frequency and sleep-wakefulness cycle in this fish species further substantiates the hypothesis that the fundamental aspects of sleep have been preserved throughout vertebrate evolution, with modifications tailored to the life history of each species.

## Electronic Supplementary Material

Below is the link to the electronic supplementary material.

Supplementary Material 1: Movie_1.mp4. This video was used in the preparation of Fig 2, showing ventilation (opercular expansion—compression) and yawning.

Supplementary Material 2: Movie_2.mp4. This video was used in the preparation of Fig 5, showing waving activity. It is important to note that the playback speed of this video is increased by a factor of ten.

Supplementary Material 3: Movie_3.mp4. This video was used in the preparation of Fig 6, showing a rapid eye movement (REM) episode.

## Data Availability

The datasets for the current study are available from the corresponding author on reasonable request.
